# Strategies to improve the care of older adults 50 years and above living with HIV in Uganda

**DOI:** 10.1186/s12981-023-00550-y

**Published:** 2023-11-04

**Authors:** Scovia Nalugo Mbalinda, Derrick Amooti Lusota, Martin Muddu, David Musoke, Mathew Nyashanu

**Affiliations:** 1 Department of Nursing, College of Health Sciences, School of Health Sciences, Makerere University, P.O. Box 7072, Kampala, Uganda; 2 Makerere University Joint AIDS Program (MJAP), Kampala, Uganda; 3 Department of Disease Control and Environmental Health, School of Public Health, College of Health Sciences, Makerere University, Kampala, Uganda; 4 Department of Health & Allied Professions School of Social Science, Nottingham Trent University, Nottingham, UK

**Keywords:** Elderly PLHIV, Strategies, Improved care, Uganda

## Abstract

**Introduction:**

With effective antiretroviral therapy (ART), many persons living with HIV (PLHIV) live to old age. Caring for aged PLHIV necessitates the engagement of caregivers and patients to establish agreed-upon goals of treatment. However, there is limited literature on friendly and centered models of care for elderly PLHIV. We explored strategies to improve care in HIV clinics among PLHIV aged 50 years and above in Uganda.

**Methods:**

We conducted 40 in-depth interviews in two hospitals with elderly PLHIV aged 50 years and above who had lived with HIV for more than ten years. We explored strategies for improving care of elderly PLHIV at both health facility and community levels. The in-depth interviews were audio-recorded and transcribed verbatim. The thematic approach guided data analysis.

**Results:**

The elderly PLHIV suggested the following strategies to improve their care: creating geriatric clinics; increasing screening tests for non-communicable diseases in the ART clinics; community and home-based ART delivery; workshops at health facilities to provide health education on aging effectively; creating community support groups; financial assistance for the elderly PLHIV and advances in science.

**Conclusions:**

There is need to improve community HIV care especially for the elderly and social and economic support in the community. Involving the elderly PLHIV in developing strategies to improve their health goes a long way to improve the patients' quality of care. There is a need to incorporate the raised strategies in HIV care or older adults.

## Introduction

People living with HIV (PLHIV) have always found it challenging to cope with the condition while negating the impact of stigma that comes with it [2].Furthermore, coping with HIV comes with challenges as people grow older. It is also important to acknowledge that despite the growing number of older people living with HIV across the world, few health systems are prepared to support older PLHIV [17]. More importantly, there are limited specific transitional arrangements from adults HIV care to Geriatric HIV care in many health systems [14]. This is more pronounced and impactful in developing countries where health systems are underfunded or overwhelmed by the growing population supported by a very weak health infrastructure [18]. In Uganda ART clinics for older PLWHIV are held alongside other adults without any specialized services contextual to older people. This one size fit all approach in the care of PLWHIV has the potential to ignore some of the key issues older people living with HIV are experiencing, thereby having a negative outcome on their health outcomes. The only specific HIV special clinic held separately is the one for children and adolescence ultimately showing a gap in the care for older people living with HIV.

Health systems without specific plans and strategies to cater for the growing number of older PLHIV are risking a potential disaster taking into consideration that many of the older people are living with comorbidities such as cardiovascular disease, hypertension, type-2 diabetes, obesity, chronic kidney disease, bone disease, hyperlipidemia, cancer, depression, and neurocognitive impairment [1]. In the absence of government support services, some communities have already started informal initiatives to support older people living with HIV [13]. However, these community initiatives are hampered by poor financial support and planning literacy [26].

In many low income countries, there are many problems that are hampering the development of robust geriatric HIV care systems [14]. Such problems include low health budgets from governments and few clinics in many remote parts of low income countries [21]. Considering these problems, i due diligence should be factored into the budgets of low-income countries to make sure that the health ministries receive higher funding to make sure that more geriatric HIV clinics are built in remote parts of the countries alongside training of competent health personnel. To make this a success the governments to engage communities and make sure that the development of HIV health services is also supported by the needs of the community to enhance sustainability.

Many communities are unable to cater for the needs of older PLHIV due to other secondary engagements [23]. Such engagements include the working pattern dynamics such as living away from family due to searching for available employment. Many older people living with HIV are most likely to be living alone and experiencing loneliness [12]. This has also compounded the problems being experienced by older people living with HIV. In many circumstances, this has resulted in many older people living with HIV to miss clinical appointments and relapsing due to poor support systems within communities [8]

Many health systems across the world have been developed through the evidence provided by health professionals and politicians [11]. This has led to the marginalization of communities’ voices in health development. Furthermore, this has also led to many people within communities to shun the use of health services because of being marginalized. It is therefore important that the development of initiatives to support older people living with HIV considers their needs and aspirations to make sure that they are fully engaged. Such an approach is likely to increase service utilization as well as enhance sustainability of interventions.

HIV is a lifelong condition that is unique when presenting to different people [29]. It is also important to acknowledge that raising awareness and knowledge in communities about a condition such as HIV is an effective way of empowering the affected people alongside communities. As such, there is need to raise awareness in communities regarding any new information to make sure that older people living with HIV are constantly supported and cared for effectively. Available literature suggests that there is low knowledge about caring for PLHIV in communities making it difficult to positively improve their health outcomes [22].This study therefore explored strategies to improve the care of adults 50 years and above living with HIV in Uganda**.**

## Methods

### Study design

This was an exploratory qualitative study.

### Study setting

Participants were recruited from one district hospital and one regional referral hospital. Both hospitals were public health facilities. The health facilities offered HIV services, including voluntary counselling and testing (VCT), prevention of mother-to-child transmission (PMTCT), voluntary medical male circumcision (VMMC), provision of antiretroviral therapy (ART), and the management of HIV-related comorbidities including TB, cardiovascular disease, chronic kidney disease, osteopenia, osteoporosis, hepatic disease, and cancer.

### Sampling strategy

Participants were purposively selected from the public health facilities. The participants were 50 years old and above and had been on ART treatment for ten years. Both males and females were selected to be interviewed.

### Participant selection

The researchers recruited 40 participants from August to September 2020. Participants were purposively selected from the public health facilities. The inclusion criterion was a willingness to participate in the study, and participants were 50 years and above. The participants had also been on ART treatment for ten years or more. Participants gave individual written consent before taking part in the study. The researchers explained the purpose of the study and sought the consent of participants to take part.

### Data collection procedures

The tool development was informed by existing literature. Before data collection, an in-depth interview guide was pretested among older patients living with HIV in another clinic. Socio-demographic characteristics were obtained from study participants. The study employed semi-structured in-depth interviews (IDIs) to collect data to understand strategies to improve care in HIV clinics among PLHIV aged 50 years and above in Uganda. We explored issues on access to resources to improve their livelihood and care in the clinic as they age. The research team conducted IDIs in a private meeting room at the health facility. Each IDI lasted 45 min to 1 h. The lead researcher who has experience in conducting qualitative studies for the last ten years conducted the IDIs. An interview guide with open-ended and probing questions was used to collect data. Participants decided whether to take the interview in English or the local language (Luganda). All interviews were audio recorded. Data collection stopped when no significant new information emerged from interaction with the participants.

### Data management and analysis

Data were transcribed verbatim, and transcripts were returned to participants for comments and corrections. A language expert translated the approved transcripts that were in the local language into English. Two researchers coded the data independently, identified and highlighted concepts and key phrases, and obtained emerging themes. The codes were aggregated into themes (groups of word patterns or phrases with similar meanings) to describe the strategies to improve care in HIV clinics among PLHIV aged 50 years and above in Uganda. QSR International Pty Ltd. (2018) NVivo (Version 12), https://www.qsrinternational.com/nvivo-qualitative-data-analysis-software/homeNVivo 10, a software package, was used to manage the data. Data analysis involved the evaluation of audio recordings and field notes to transcribe the data. The transcripts were analyzed thematically as described by Mays and Pope [15]. Data analysis employed the constant comparative method to analyze codes or meaning units (recurrent patterns statements, words or phrases with similar meaning or interpretation) across the data set [4, 28]. Representative quotes from participants derived from the individual transcripts were included to illustrate the source of interpretations of information. The researchers were careful to avoid compressing the data too much to keep the findings’ richness and distinctiveness. All text excerpts were de-identified. A sample of four participants provided feedback on the findings to enhance the trustworthiness and credibility of the data analysis.

### Ethical considerations

Permission to conduct the study was obtained from the Makerere University School of Health Science Research and Ethics committee (MAKSHSREC-2021-104) and the Uganda National Council for Science and Technology (HS1759ES). All participants gave written informed consent to participate. The consent was obtained at the time of their ART Refill. Any information shared about unpleasant experiences from previous experiences was discussed in an empathetic manner. Participantss who seemed emotionally affected by the recall of unpleasant experiences were counselled. Participants were assured that they were free to participate and that even if they declined, their decision would not affect the due care that they were entitled to.

## Results

### Participants’ characteristics

Participants were aged 50–71 years. The majority, 20 (50%), were married, and all of them had spent ten years and more on ART and said to have acquired HIV through sexual intercourse (Table 1).

**Table 1:** Socio-demographic characteristics of the participants

Variable	Frequency (N = 40)	Percentage (%)
Age categories (years)		
50–54	17	42.5
55–59	11	27.5
60–64	8	20
65–69	3	7.5
70–74	1	2.5
Sex		
Male	19	47.5
Female	21	52.5
Marital status		
Married	20	50.0
Single	2	5.0
Divorced/separated	9	22.5
Widowed	9	22.5
Religion		
Catholic	26	65.0
Anglican	9	22.5
Muslim	2	5.0
Born again	3	7.5
Highest level of education		
None	5	12.5
Primary	18	45.0
Secondary	9	22.5
Tertiary/university	8	20.0
Employment status		
Employed	30	75.0
Unemployed	10	25.0
Number of children		
0–4	23	57.5
5–9	14	35.0
10–14	2	5.0.0
15–19	1	2.5.0
Time since HIV diagnosis in years		
10–14	18	45.0
15–19	18	45.0
20–24	4	10.0
Time since ART initiation		
10–14	21	52.5
15–19	17	42.5
20–24	2	5.0
How were you infected with HIV?		
Unprotected sex	40	100

The elderly PLHIV suggested the following strategies to improve their care: creating geriatric clinics; increasing screening tests for non-communicable diseases in the ART clinics; community and home-based ART delivery; workshops at health facilities to provide health education on aging effectively; creating community support groups; and financial assistance for the elderly PLHIV and advances in science.

### Creating geriatric clinics

The participants expressed interest in having a special private place at the health facility where their needs would be addressed. Furthermore, they asserted that such a place could enable them to discuss some issues which are normally hard to discuss in a clinic that encompasses all ages. This is seen below:

“sharing ideas with people of your age is also helpful. But now if you come; you are here and your age mate is the other side, you may not be able to talk and exchange ideas. Your age mate may be suffering from the same disease like the one you have and they tell you how they are managing theirs,………. So I am suggesting that they should give us a special room specifically for the old persons such that when someone comes, they know that is our special room where I go to when I go to the clinic. Like patients of pressure, diabetes, pregnant women or adolescents have special room” 67 year old male.

“What I can say, there should be special days for the elderly, like there is special days for the youths, and those of children. To me I think if we are given special days let’s say today, we have the elderly, that means that the information given that day will target the elderly’’ 56-year-old female.

### Increasing screening tests for non-communicable diseases in the ART clinics

The participants acknowledged that they are always tested for issues related to HIV, but they rarely test for other chronic diseases such as diabetes. Many of them reported knowing about other conditions affecting them very late.

“The government should provide the hospitals with more machines for testing other diseases for example when someone is tested for HIV; he is also tested for any other diseases that he/she might be having in his/her body and those related to aging. So that they can know what we are suffering from and what might be its cause such that it is also treated’’ 63 year old female.

“Personally I didn’t know that I had diabetes until I collapsed in the clinic one day……….if they had tested me earlier may be I wouldn’t collapse’’ 56 year old male.

### Community and home-based ART delivery

The participants felt that services should be taken closer to them because as they age, they might not have energy and transport to move to their respective clinics. Furthermore, they suggested that home delivery of medication can help them solve the problem of travelling to HIV clinics for medication.

“, if I am weak and I am unable to come here, health workers to bring me medicine…. If I grow old and I am unable to come, I can call them and let them know so that they bring my medication or send someone I have registered to pick for me the medication. That’s what I want to have good communication with the health workers” 60-year-old female.

“Health workers should bring the medicine at our homes, bring you some eats at home; that’s what I hope in the future when I am old, I cannot work, or afford, they come to my home, bring me medicine, sugar, cooking old when I can’t get them”. 66 year old female.

### Workshops at health facilities to provide health education on aging effectively

To both working and non-working participants, holding a job was healthful. They mentioned that having something to do to generate income not only engaged them temporally, mentally, physically, and economically but also contributed to their self-worth. The participants acknowledged that they need some workshops to address issues not only related to health but also address issues of survival and financial issues.

“So I would put in this request for workshops, on good diet, about financial management, guidance and counseling. Like though we have aged but we would love to learn more about financial management. Leave out only starting Savings and Credit Cooperative Organisation or Society (SACCOs) but we should also learn how to manage our finances’’. 53-year-old female.

### Creating community support groups

The participants acknowledged the need for support groups that can help the vulnerable in the community.

“I would love for government to look after us as their own old people…………………. Like giving us a salary! You hear some people getting salary at the end of the month, government can do that. Now you may not have soap, you know that you will get some money from government and buy it and or giving us food”.

“The leaders, they would endeavor to support someone who has grown because he/she can be there when they do not have any kind of support. Even in the hospital here, they should support people who have grown. May be setting up for them something they can do, or getting them what to eat, getting them soap because there are old people who cannot afford those” (60 year old male).

#### Financial assistance

The research participants expressed that they needed some help with financial assistance to enable them meeting some of their health needs.

I wish we could have support groups in the community to support those who are vulnerable like I have a poorly constructed toilet, even the diseases I would have prevented I end up getting them which might affect my health. Secondly if there is a way, I can get a new wheel chair because this one I have is all spoilt. So, if there is any way I can get a new one I will be grateful for it”. (68 year old male).

### Advances in health care

The participants in this study had been on ART for more than 10 years and probably they are fatigued with the daily intake of medication. They wish that scientists could come up with regimens that they can take at least once in 6 months.

“Ok like we are taking this medicine, I wish they make us medicine for an injection. You come here may be give you an injection for three months or 6 months” (57 year old female)

“I would suggest that government can get us injections. Someone is given an injection may be like for 6 months, and we stopping taking this daily medicine” (65 year old male).Let’s get the injection so that people do not have to move to come here pick the daily medicine.

## Discussion

We explored strategies for improving care of elderly PLHIV at both health facility and community levels. The elderly PLHIV suggested the following strategies to improve their care: creating geriatric clinics; increasing screening tests for non-communicable diseases in the ART clinics; community and home-based ART delivery; workshops at health facilities to provide health education on aging effectively; creating community support groups; financial assistance for the elderly PLHIV and advances in science.

A specialist clinic dealing with common problems for a group of people living with a lifelong condition can empower them to discuss their problems [27]. Such a clinic can also act as a confidential safety net compared to a generic clinic that caters for people with different age groups and conditions. In this study, research participants who were elderly people with HIV expressed interest in having a special place at health facilities catering for their aging problems. Furthermore, they asserted that such a room can enable them to discuss some issues which are normally hard to discuss in a clinic that encompasses all ages. Considering the above finding, health facilities through the Ministry of Health should develop specialist clinics that can offer a safe and confidential space for older people living with HIV to discuss their concerns [9]. Furthermore, such clinics need to be supported by health professionals who understand the needs of older people apart from understanding the treatment and management of HIV.

Many older people living with HIV came to know about other conditions affecting them later than expected thereby impacting on their health and well-being [10]. This is also compounded by the fact that many older people perceive coming to the HIV clinic are normally tested for HIV and ignore other conditions that are associated with older age [20]. In this study, the participants acknowledged that they were always tested for issues related to HIV, but rarely test for other chronic diseases such as diabetes. Many of them reported knowing about other conditions affecting them very late. Considering the above finding, it is therefore important that geriatric HIV clinics should also be proactive in testing other conditions apart from HIV that may affect older people. This will provide an opportunity to quickly identify other issues that may be affecting older people living with HIV thereby enabling quick treatment and positive health outcomes.

During the COVID-19 pandemic, many people living with HIV ran out of their medication and found it very difficult to get supplies [19]. This caused some people to relapse and presented late at treatment facilities when they were very sick [6, 19]. Furthermore, many older people living with HIV find it difficult to get money for transport to collect their medicine supplies at the HIV clinics. In this study, the participants felt that services should be brought closer to them as they age because they might not have energy and transport to move to their respective clinics. Furthermore, they suggested that home delivery of medication can help them solve the problem of travelling to HIV clinics for medication. It is therefore important for the central government through the Ministry of Health to consider erecting HIV clinics within the neighborhoods of older people living with HIV and medicine home delivery for older people living with HIV. Such an initiative will make sure that older people living with HIV will not run out of medication like what happened during the COVID-19 pandemic [19]. Furthermore, it will prevent older people living with HIV from running out of medicines and relapsing.

Raising awareness of health and other life issues among people living with a lifelong conditions like HIV or diabetes is associated with positive health outcomes on the affected individuals [3]. Furthermore, it can also help them to quickly seek for help or treatment once they are affected by the problems [24]. In this study, participants acknowledged that they need some workshops to address issues not only related to health but also address issues of survival and financial issues. Considering the above finding, the central government through the ministry of labor and manpower establish training of life skills and provide services information to older people living with HIV. Such training can be rolled out in communities through community health workers who are normally in contact with older people living with HIV.

Support groups and financial assistance for people living with lifelong conditions like HIV and diabetes is very important in improving their health and well-being [25]. Such groups and assistance can prevent them from loneliness and poverty normally associated with older people in many low- and middle-income countries (LMICs) [16]. In this study, participants acknowledged the need for support groups that can help the vulnerable in the community and wished the health workers, local leaders as well as the government to help them in terms of health and financial assistance. Looking at the above findings the central government through the ministry of health should help communities to form support groups for older people living with HIV and provide poverty alleviation grants to struggling older people living with HIV. Such initiatives can improve the health outcomes of older people living with HIV.

Daily intake of medication can be tiresome to an individual especially when the number of tablets is high [7]. Furthermore, in many LMICs the tablets used to treat chronic conditions are normally the old generations which are sometimes heavy for people taking them [5]. In this study, participants wished that scientists could come up with advanced treatment regimens that they can take at least once in 6 months. Considering the above finding there is need for developed countries to support LMICs to secure modern medication which has a light consumption rate and high treatment output. Such medications can make it easy for older people living with HIV to easily cope with their medication.

### Limitations and strengths of the study

The study was carried out in two district of Uganda therefore the findings may not be generalizable to other parts of the country. Research encompassing more regions across sub-Sahara Africa may be necessary to understand the strategies to improve the care of adults 50 years and above living with HIV across the continent. The study utilized a qualitative paradigm. Another study utilizing mixed methods may be necessary to enable the exploration of strategies to improve the care of adults 50 years and above living with HIV from different ontological and epistemological positions.

## Conclusion

Informed strategies to improve the care of adults 50 years and above living with HIV is important to enhance ongoing improvements in HIV treatment and care. More importantly, robust policies supporting HIV education and awareness are needed to make sure that the public are aware of the need to support adults living with HIV.

## Data Availability

The acquired and/or analyzed data are not publicly available because of the lack of authorisation from the children’s legal guardians, and the agreement with the Research Ethics Committee that the database would remain with the corresponding author only. However, all data can be made available by the corresponding author upon reasonable request.

## Abbreviations

ART: Anti retro viral therapy HIV human immunodeficiency virus
MOH: Ministry of health
HAART: Highly active antiretroviral therapy
PLHIV: Persons living with HIV
